# What contributes most to the SPPB and its subscores in hospitalized geriatric patients: an ICF model-based approach

**DOI:** 10.1186/s12877-022-03358-z

**Published:** 2022-08-13

**Authors:** Jennifer Kudelka, Johanna Geritz, Julius Welzel, Hanna Hildesheim, Corina Maetzler, Kirsten Emmert, Katharina Niemann, Markus A. Hobert, Andrea Pilotto, Philipp Bergmann, Walter Maetzler

**Affiliations:** 1 Department of Neurology, University Hospital Schleswig-Holstein, Arnold-Heller-Straße 3, 24105 Kiel, Germany; 2 Neurology Unit, Department of Clinical and Experimental Sciences, University of Brescia, P.le Spedali Civili 1, 25123 Brescia, Italy; 3 Department of Internal Medicine I, University Hospital Schleswig-Holstein, Arnold-Heller-Straße 3, 24105 Kiel, Germany

**Keywords:** Fear of falling, Grip strength, Mobility deficits, International classification of functioning, disability and health, Comprehensive geriatric assessment

## Abstract

**Background:**

Mobility deficits are highly prevalent among geriatric patients and have serious impact on quality of life, hospitalizations, and mortality. This study aims to capture predictors of mobility deficits in hospitalized geriatric patients using the International Classification of Functioning, Disability and Health (ICF) model as a framework.

**Methods:**

Data were obtained from *n* = 397 patients (78 ± 7 years, 15 ± 7 ICD-11 diagnoses) on a geriatric ward at time of admission. Mobility was assessed using the Short Physical Performance Battery (SPPB) total score and gait, static balance and transfer subscores. Parameters from an extensive assessment including medical history, neuropsychological and motor examination, and questionnaires were assigned to the five components of the ICF model. Spearman’s Correlation and multiple linear regression analyses were calculated to identify predictors for the SPPB total score and subscores.

**Results:**

Use of walking aid, fear of falling (FOF, but not occurrence of previous falls), participation in society, ADL and grip strength were strongly associated with the SPPB total score and all subscores (*p* < .001). FOF and grip strength were significant predictors for the SPPB total score as well as for gait and transfer subscores. FOF also showed a strong association with the static balance subscore. The clinical parameters of the ICF model could only partially explain the variance in the SPPB total score (24%) and subscores (12–23%), with no parameter from the activities and participation component being significantly predictive.

**Conclusions:**

FOF and reduced grip strength are associated with mobility deficits in a hospitalized geriatric cohort. Further research should focus on interventions to reduce FOF and increase muscle strength in geriatric patients. Moreover, there is a need for ICF-based assessments instruments (especially in the activities and participation components) that allow a holistic view on mobility and further daily life-relevant health aspects in geriatric patients.

## Background

Due to demographic change, the proportion of older people and people with age-related multimorbidity in the overall population is increasing [1–3]. This development has serious consequences for those affected, but also for health policy. One of the most common health problems of geriatric patients are mobility deficits, mainly based on gait and balance disorders, which have a prevalence of 35% in adults older than 70 years [4]. Gait and balance disorders can have, among others, neurological, internal or mechanical causes and are mostly based on various diseases as part of a multimorbidity [5–7]. Common neurological causes of gait and balance disorders include polyneuropathy, neurodegenerative diseases such as Parkinson’s disease (PD), and cognitive deficits [5, 8–10]. In addition, sarcopenia and frailty have been found to play an important role in mobility deficits, for example by increasing the risk of falls [11–13].

The Short Physical Performance Battery [14] (SPPB) is a commonly used tool to assess mobility in community-dwelling as well as in-hospital older adults and has proven high test-retest reliability and predictive validity [15, 16]. Poor mobility measured by the SPPB has shown to be associated with decreased quality of life [17], disability in daily activities [18], risk of falls [19], hospitalization [20] and mortality [21].

In 2001, the World Health Organization (WHO) established the International Classification of Disability, Functioning and Health (ICF) [22] as an addition to the International Statistical Classification of Diseases and Related Health Problems (ICD-11) [23]. In contrast to the ICD-11, the ICF does not focus on the etiology of the health problem, but on the functional ability and disability of the person affected. Due to the different perspectives that both classifications cast on a health problem, they should be understood as mutual complements for a more holistic classification of health conditions. The ICF model provides a suitable framework to conceptualize certain determinants of health [24, 25], e.g. mobility deficits. It defines three different components of functioning and disability (body functions and structure, activities, and participation) as well as two contextual factors (environmental and personal factors). Thus, the ICF understands body functions and structure as anatomical and physiological entities such as organ systems, but also mental and spiritual body functions, which result in damage to the organism regardless of their pathology. The activities and participation components, on the other hand, classify the patient’s ability to perform daily activities and participate in social and community life. The environmental and personal factors describe the patient’s life background, e.g. the domestic environment, society and cultural structures, age, sex, education, and personal experiences [22].

This paper addresses the question to what extent mobility and its individual components, such as gait, static balance and transfer can be predicted using the ICF model as a framework and which parameters are suitable for predicting mobility deficits in a vulnerable and multimorbid geriatric cohort.

## Methods

### Study design

Data were obtained from the multicenter study “COgnitive and Motor interactions in Older populatioNs” (ComOn) [26]. ComOn is a prospective exploratory study that assesses balance, gait and cognition, among other parameters of a quantitatively oriented comprehensive geriatric assessment, in hospitalized geriatric patients. The study was approved by the ethics committee of the Medical Faculty of the University of Kiel (D427/17) and conducted according to the principles of the Declaration of Helsinki (version of 2013). All participants or their legal guardians provided informed consent before taking part in this study.

### Participants

**Fig. 1:**
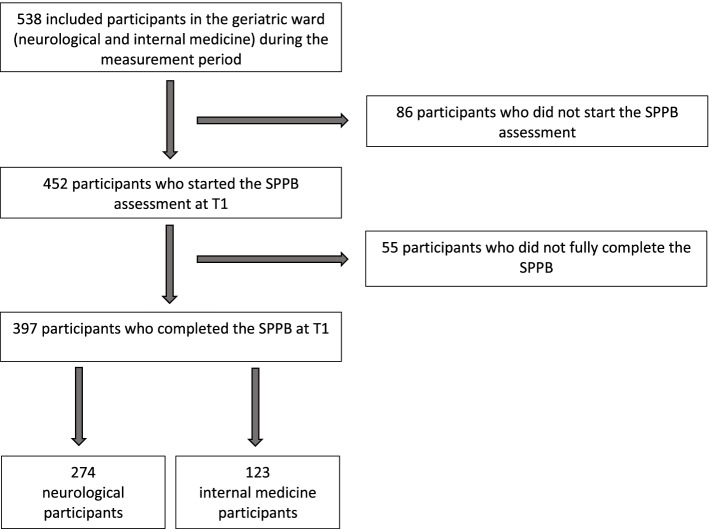
Process of inclusion and exclusion of data sets collected during the measurement period in the statistical analyses. SPPB = Short Physical Performance Battery

Participants were recruited from an interdisciplinary neurological and internal medicine geriatric ward. Detailed information about in- and exclusion criteria is reported elsewhere [26]. Briefly, participants were included if they were > 70 years of age or were multimorbid > 5 years, had at least one chronic condition, and scored at least 5 points in the Montreal Cognitive Assessment (MoCA) [27]. Moreover, the ability to walk at least 4 meters with or without walking aids was required for inclusion. After obtaining informed consent and being screened for eligibility criteria, participants were examined within the first 2 days of their inpatient stay. The assessment included the collection of demographic and medical data as well as the conduct of a comprehensive neuropsychological and motor examination. Participants were given questionnaires to complete during their inpatient stay (see below). The process of inclusion and exclusion of data sets collected during the measurement period is displayed in Fig. 1.

### Assessment of mobility

Mobility was assessed using the SPPB, which consists of the total score and the three subscores gait, static balance and transfer. For the assessment of static balance, three positions (side-by-side, semi tandem and tandem stand) have to be held in free standing for at least 10 seconds. The gait test is performed by asking the participant to walk a distance of 4 meters twice in their usual walking speed with or without using walking aids. The faster walk is used for scoring. In the sit-to-stand test, the participant is asked to stand up from the chair and sit down again as quickly as possible 5 times in a row. For all subscores, 0 to 4 points can be awarded, so that the SPPB total score ranges from 0 to 12 points, with a higher score representing better mobility.

### ICF parameters

Parameters of the comprehensive geriatric assessment and demographic and medical data were categorized into the components and contextual factors of the ICF model: Number of ICD-11 diagnoses (as a measure of comorbidity), grip strength, motor symptoms, global cognition, depressive symptoms, fear of falling (FOF) and pain were assigned to the ICF body functions and structure component. Grip strength was assessed using the highest score of 4 attempts (2 times with each hand) measured by the Jamar hydraulic hand dynamometer [28] (Lafayette Instrument Company, Lafayette, USA). The Movement Disorder Society-revised Unified PD Rating Scale (MDS-UPDRS) III [29] was used to assess motor symptoms (in PD patients during medication ON phase). The MDS-UPDRS III is a commonly used tool in PD, but can also be used to measure motor symptoms in geriatric patients with other conditions [30]. The MoCA [27] was used to measure global cognition. Depressive symptoms were evaluated using the *Depression-im-Alter* scale (DIA-S) [31], which was especially developed for geriatric patients. FOF was assessed using the total score of the Falls Efficacy Scale (FES-I) [32], in which participants have to indicate how concerned they are about falling when performing 16 different activities. The Lachs Geriatric Screening [33] (question 15) was used to record if participants often suffer from pain.

Activities of daily living [34] (ADL, e.g. bathing, dressing), instrumental activities of daily living [35] (IADL, e.g. food preparation, housekeeping) and daily function were included in the ICF activities component. ADL and IADL were assessed during the medical history interview, which was conducted during the first 2 days of the inpatient stay. Daily function was assessed using the Barthel index [36], which is a widely established tool, especially in geriatric rehabilitation patients, with high structural validity, reliability and interpretability [37]. The Barthel index was collected by the nursing staff during the first 2 days of the hospital stay and includes 10 basic daily activities. Measures assigned to the participation component were social activities, participation in society and living conditions (alone vs. not alone). Five questions (questions 2, 4, 11, 14, 15) from the *Nürnberger-Alters-Alltagsskala* (NAA) [38] were used to assess how often participants performed social activities. Participation in society was evaluated using two items of the FES-I [32] (question 12: visiting a friend or relative; question 16: going out to a social event). Whether participants lived alone or not was asked during the medical history interview. Place of residence (nursing home vs. own home) and use of walking aid were assigned to the environmental factors. Age, sex, years of education and occurrence of falls in the last three months (assessed by the Lachs Geriatric Screening [33], question 13) were classified as personal factors.

### Statistical analysis

Data were analyzed using the statistical software JASP [39] (version 0.16.0.0) and presented as mean and standard deviation for metric scaled parameters and percentage of the total for dichotomous parameters. Missing values were replaced in single items by individual mean imputation if questionnaires were self-reported, showed high internal consistency and completeness of at least 80% [40]. This affected 38 FES-I datasets (29 missing one value, completeness of 94%; 9 missing two values, completeness of 88%). In a first step, the individual parameters were correlated with each other and with the SPPB total score and the subscores according to Spearman’s rho coefficient (*ρ*). In case of correlations of *ρ* > 0.4 or *ρ* < − 0.4, one of the parameters was removed from the regression analyses in order to minimize multicollinearity [41]. Parameters were also excluded from the model that did not show significant correlation (*p ≥* .05) with the dependent variable (total SPPB score or subscores). In a second step, four multiple linear regression analyses (using forced entry method) were calculated using the SPPB total score and the subscores as dependent variables. Significance level was set at (adjusted) *p* < 0.05. To avoid the variance in the model from being significantly determined by the number of parameters, the adjusted R^2^ was used as an indicator for the goodness of model fit of the overall model. Moreover, the standardized regression coefficient β was calculated for each parameter, describing the weighting of the parameter in the overall model.

## Results

### Descriptive data

Of the 397 participants included in these analyses, 49.4% were women. The cohort was aged 78 ± 7 years. A walking aid was used by 51.8% participants, and 51.5% had fallen at least once in the past 3 months. Participants scored 6 ± 2 points in the total SPPB. SPPB subscores were higher for gait and static balance than for transfer. Participants had, as a mean, 15 ± 7 ICD-11 diagnoses. Diagnoses within the urogenital (314 participants with at least one diagnosis), circulatory (304), neurological (289), and endocrine, nutritional and metabolic systems (274) were most common. Most common neurological disorders were PD (119 participants affected) polyneuropathy (91), and cerebrovascular disease (85). Delirium was rare (19). Table 1 provides further details.

**Table 1:** Demographic and clinical data

Parameter	ICF	n	M (SD)	[Min; Max]
Age [years]	PERS	397	78 (7)	[51; 97]
Education [years]	PERS	378	10 (2)	[6; 22]
Sex° [n, %]	PERS	397	female	196 (49.4%)
			male	201 (50.6%)
Place of residence° [n, %]	ENV	389	nursing home	33 (8.5%)
			own home	356 (91.5%)
Living conditions° [n, %]	PART	353	alone	138 (39.1%)
			not alone	215 (60.9%)
Comorbidities (ICD-11 diagnoses)	BODY	384	15 (7)	[1; 35]
Grip strength [kg]	BODY	381	24 (10)	[3; 54]
Motor symptoms (MDS-UPDRS III, 0–132 points)	BODY	328	25 (15)	[1; 74]
Global cognition (MoCA, 0–30 points)	BODY	363	21 (4)	[7; 30]
Depressive symptoms (DIA-S, 0–10 points)	BODY	346	2.8 (2.5)	[0; 10]
FOF (FES-I, 16–64 points)	BODY	318	33 (12)	[16; 64]
ADL (0–6 points)	ACT	386	5.0 (1.0)	[1; 6]
IADL (0–8 points)	ACT	277	6.3 (1.8)	[1; 8]
Daily function (Barthel index, 0–100 points)	ACT	362	57 (13)	[20; 100]
Social activities (NAA subscore, 5–15 points)	PART	305	9.3 (2.0)	[5; 15]
Participation in society (FES-I subscore, 2–8 points)	PART	309	3.7 (1.9)	[2; 8]
Pain° [n, %]	BODY	377	yes	180 (47.7%)
			no	197 (52.3%)
Walking aid° [n, %]	ENV	361	yes	187 (51.8%)
			no	174 (48.2%)
Occurrence of falls in the last three months° [n, %]	PERS	388	yes	200 (51.5%)
			no	188 (48.5%)
SPPB total score		397	5.5 (2.1)	[0; 11]
SPPB gait subscore		397	2.1 (0.9)	[0; 4]
SPPB static balance subscore		397	2.5 (1.2)	[0; 4]
SPPB transfer subscore		397	1.0 (0.7)	[0; 4]

Metric scaled data are presented with number of participants (n), mean value (M), standard deviation (SD), and range (min; max). Dichotomous data (°) are shown with number of participants (n) and percentages (%)

*ACT* Activities, ADL Activities of daily living, *BODY* Body functions and structure, *DIA-S Depression-im-Alter* scale, *ENV* Environmental factors, *FES-I* Falls Efficacy Scale-International, *FOF* Fear of falling, *IADL* Instrumental activities of daily living, *ICD* International Statistical Classification of Diseases and Related Health Problems, *ICF* International Classification of Functioning, Disability and Health, *MDS-UPDRS* Movement Disorder Society-revised Unified Parkinson’s Disease Rating Scale, *MoCA* Montreal Cognitive Assessment, *NAA Nürnberger-Alters-Alltagsskala*, *PART* Participation, *PERS* Personal factors, *SPPB* Short Physical Performance Battery

### Correlation analyses

**Fig. 2:**
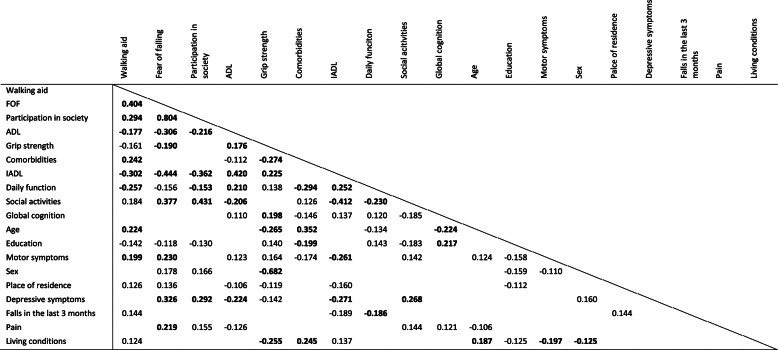
Correlation analysis between all parameters included in the International Classification of Functioning, Disability and Health (ICF) model. Correlations are displayed using Spearman’s rho (ρ). Correlations with *p* < .001 are marked bold. Non-significant correlations (*p* ≥ .05) are not shown. ADL = activities of daily living, FOF = fear of falling, IADL = instrumental activities of daily living

The results of the Spearman correlation analysis are presented in Table 2. All parameters included in the analysis were correlated with the total SPPB score and the SPPB subscores gait, static balance, and transfer. The highest linear correlations were found between the SPPB total score and the use of a walking aid (*ρ* = − 0.481), FOF (*ρ* = − 0.437), high participation in society (*ρ* = − 0.327), high performance in ADL (*ρ* = 0.298), IADL (*ρ* = 0.251), daily function (*ρ* = 0.236), and social activities (*ρ* = − 0.222), high grip strength (*ρ* = 0.284), less comorbidities (*ρ* = − 0.258), high level of global cognition (*ρ* = 0.211), more years of education (*ρ* = 0.194), and younger age (*ρ* = − 0.198). Parameters that showed the strongest correlation with the SPPB total score and all subscores (gait, static balance, transfer) were use of walking aid, FOF, participation in society, ADL, and grip strength. Living conditions, place of residence, pain, occurrence of falls in the last 3 months, and depressive symptoms only showed weak or no correlations with the SPPB total scores and all subscores. Correlation analyses of the parameters with each other were performed as well (see Fig. 2). Due to multicollinearity, IADL (*ρ* > 0.4 with FOF, ADL and social activities), participation in society (*ρ* > 0.4 with FOF and social activities), walking aid (*ρ* > 0.4 with FOF), and sex (*ρ* > 0.4 with grip strength) were removed from the regression model. Note that in a post-hoc multiple linear regression analysis with all alternative regression models, walking aid (instead of FOF) was also significantly associated with the SPPB total score, but IADL, participation in society and sex were not.

**Table 2:** Correlation analyses with Short Physical Performance Battery (SPPB) total score and subscores

Parameter	ICF	SPPB total	Gait	Static balance	Transfer
Walking aid	ENV	−0.481***	−0.392***	−0.391***	−0.237***
FOF (FES-I)	BODY	−0.437***	− 0.327***	− 0.339***	− 0.294***
Participation in society (FES-I)	PART	− 0.327***	− 0.278***	− 0.249***	− 0.199***
ADL	ACT	0.298***	0.216***	0.243***	0.223***
Grip strength	BODY	0.284***	0.199***	0.238***	0.216***
Comorbidities (ICD-11 diagnoses)	BODY	−0.258***	−0.271***	−0.170**	− 0.138**
IADL	ACT	0.251***	0.213***	0.190**	0.188**
Daily function (Barthel index)	ACT	0.236***	0.164**	0.183***	0.179***
Social activities (NAA)	PART	−0.222***	−0.216***	−0.122**	− 0.160**
Global cognition (MoCA)	BODY	0.211***	0.193***	0.180***	
Age	PERS	−0.198***	−0.194***	− 0.181***	
Education years	PERS	0.194***	0.164**	0.186***	
Motor symptoms (MDS-UPDRS III)	BODY	−0.172**	− 0.143**		− 0.159**
Sex	PERS	−0.135**	−0.108*	− 0.145**	
Place of residence	ENV	−0.130*		−0.163**	
Depressive symptoms (DIA-S)	BODY	−0.113*		−0.121*	
Fall in the last 3 months	PERS	−0.103*	−0.119*		
Pain	BODY		−0.109*		−0.118*
Living conditions	PART				

Correlations are displayed using Spearman’s rho (*ρ*). Correlations with *p* < .001 are marked ***, *p* < .01 are marked **, and *p* < .05 are marked *. Non-significant correlations are not shown

*ACT* Activities, *ADL* Activities of daily living, *BODY* Body functions and structure, *DIA-S Depression-im-Alter* scale, *ENV* Environmental factors, *FES-I* Falls Efficacy Scale-International, *FOF* Fear of falling, *IADL* Instrumental activities of daily living, *ICD* International Statistical Classification of Diseases and Related Health Problems, *ICF* International Classification of Functioning, Disability and Health, *MDS-UPDRS* Movement Disorder Society-revised Unified Parkinson’s Disease Rating Scale, *NAA Nürnberger-Alters-Alltagsskala*, *PART* Participation, *PERS* Personal factors, *SPPB* Short Physical Performance Battery

### Multiple regression analyses

**Fig. 3:**
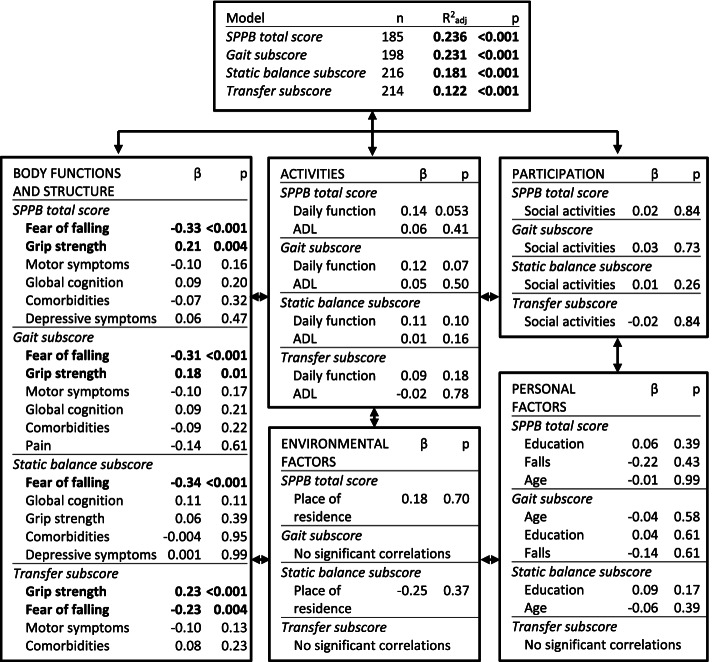
Multiple regression analyses based on the International Classification of Functioning, Disability and Health (ICF) model. Four multiple regression analyses were performed with different dependent variables: SPPB total score, SPPB gait subscore, SPPB static balance subscore, and SPPB transfer subscore. Goodness of the model is displayed with the adjusted coefficient of determination (R^2^_adj_) and significance level (p). Independent variables are displayed with standardized regression coefficient (ß) and significance level (p). Significant results (*p* < .05) are marked bold. ADL = activities of daily living, SPPB = Short Physical Performance Battery

Multiple regression analyses were performed using the SPPB total score and the SPPB subscores gait, static balance and transfer as the dependent variables (see Fig. 3). Only complete datasets were included in the models. All models were significant (*p* < .001). 24% of the variance in the SPPB total score could be explained by the model with the SPPB total score as dependent variable (*R*^2^_adj_ = 0.24). Within the models including the SPPB subscores as dependent variables, variance in gait could be explained best (*R*^2^_adj_ = 0.23), followed by static balance (*R*^2^_adj_ = 0.18) and transfer (*R*^2^_adj_ = 0.12).

Two parameters of the ICF component body functions and structure, FOF (ß = -0.33, *p* < .001) and grip strength (ß = 0.21, *p* = .004), were significantly associated with the SPPB total score in the multiple linear regression analysis, with higher SPPB total score when participants had low FOF and high grip strength. FOF proved to be a significant predictor for all SPPB subscores, showing the strongest association with static balance (ß = -0.34, *p* < .001), followed by gait (ß = -0.31, *p* < .001) and transfer (ß = -0.23, *p* = .004). Grip strength demonstrated the highest association with transfer (ß = 0.23, *p* < .001), followed by gait (ß = 0.18, *p* = .01), and had no significant association with static balance. The association between daily function and SPPB total score showed a trend towards significance (ß = 0.14, *p* = .053). No parameter neither from the ICF components activities and participation nor from the environmental or personal contextual factors showed a significant association with the SPPB total score or the three subscores in the multiple regression analyses.

## Discussion

The aim of this paper was to evaluate the key factors linked to overall mobility and individual components of mobility such as gait, static balance and transfer in a cohort of hospitalized geriatric patients based on an ICF framework. More specifically, we aimed at identifying parameters associated with mobility deficits in this large vulnerable cohort. Correlation analyses were performed to examine the strength of the relationships among the ICF-based parameters and with the SPPB total score and SPPB subscores. In total, five parameters showed a strong correlation with the SPPB total score and all subscores: Use of walking aid, FOF, participation in society, ADL, and grip strength (see Table 2). It should be emphasized that these parameters cover all three ICF components of functioning and disability as well as environmental factors. They are therefore very well distributed across the entire ICF model. This result speaks for the usefulness of broad assessment panels for the evaluation of impairments such as mobility deficits, that are relevant to everyday life. In this way, it is possible to depict the deficits in their entire range and impact. The only ICF component not covered by these five parameters is personal factors. Among personal factors, age showed the greatest association with mobility; however, comorbidities (assigned to body functions and structure) appear to have a greater influence in this vulnerable cohort. The association of age and comorbidities with SPPB total score is consistent with other research findings [14, 42]. However, there is a need for research that addresses vulnerable, multimorbid cohorts, as to date most studies have focused on healthy community-dwelling older adults [4, 15, 19, 43, 44].

The strongest correlation was found between mobility and the use of walking aids, with participants not using a walking aid achieving higher scores in the SPPB and all subscores. This observation may simply point towards poorer physical performance in those in need of a walking aid. However, data on the effects of walking aids differ. While some studies show improved balance and mobility in walking aid users [45, 46], other studies indeed suggest that use of walking aids is associated with falls, often due to incorrect use of the walking aids [43, 46–48]. Our results encourage to consider that not only the walking aid alone, but also adequate training in its use is necessary to achieve an improvement in mobility.

Strong correlations were found between FOF with overall mobility as well as with mobility components (gait, static balance and transfer). FOF also showed the highest predictive value for mobility in the regression analyses. In contrast, occurrence of falls in the last 3 months only showed a weak association with the SPPB total score and gait, and no correlation with static balance and transfer. Moreover, there was no significant correlation between falls in the last 3 months and FOF. These results are in line with a recent systematic review of 30 studies suggesting that FOF may have a greater impact on health-related quality of life than actual falls [49]. Other studies support the idea that FOF is not a symptom that necessarily derives from previous falls, but an independent feature that can occur even in patients who have not previously fallen [49, 50]. Interestingly, Delbaere and colleagues have found that patients with low perceived fall risk (despite high physiological fall risk) show higher physical activity, have a more positive outlook on life, and participate more in society [50]. The impact of FOF also on mobility has already been shown in previous studies [51, 52]. In a systematic review including 28 original studies, it was shown that reduced physical performance and reduced health-related quality of life are important consequences of FOF [51]. The presumed mechanism behind this lies in reduced physical activity caused by FOF, which leads to reduced mobility, for example through decreasing training effects [53, 54]. For example, in a prospective cohort study of 673 community-dwelling older adults, severe activity restriction was shown to be an independent risk factor for decline in physical performance [53]. The opposite aspect, namely that mobility deficits results in FOF, has also been widely studied [44, 55]. Curcio and colleagues found in a longitudinal study of 1725 older people that the combination of reduced mobility (SPPB ≤8 points) and self-reported mobility disability carries an individual risk of 82% for developing FOF [55]. In contrast to the majority of previous literature, this study collected data from hospitalized, multimorbid patients. Despite the multiple conditions and functional limitations that are prevalent in this cohort, FOF emerged as the most important predictor. Various clinical trials have proven that, for example, tai chi, postural control exercises, cognitive behavioral therapy [56, 57] and balance training [58] are able to improve FOF. In line with previous literature, our results highlight that diagnosis and treatment of FOF is highly relevant if mobility deficits should be effectively addressed. It is uncertain if FOF can be sufficiently managed by interventions to reduce falls alone, especially in vulnerable cohorts as investigated here. Future trials in this area should therefore focus in particular on such cohorts.

The second parameter that showed a significant association with overall mobility as well as gait and transfer in the regression analyses was grip strength. Grip strength presented to be most closely linked with transfer function using the sit-to-stand test. Originally, the SPPB is an assessment tool for lower extremity function [14]. A practical guideline on sarcopenia [59] concludes that grip strength is an appropriate parameter to measure total muscle strength and is closely associated with leg strength. Other studies suggest, analogous to the data presented here, that grip strength is an important indicator of physical performance [60–62]. A longitudinal study of 1030 participants even found that grip strength is a better predictor of clinical outcomes than muscle mass [63]. Strength training and the avoidance of sarcopenia should thus play a key role in the treatment of geriatric patients in order to prevent loss of mobility. Especially for a geriatric cohort, this is important information, as the assessment of grip strength is time efficient and does not cause major burden for the patients.

It should be noted that, with 12–24% explained variance, the ICF-based regression models could only partially explain the mobility deficits found in our participants. Both significantly associated parameters (FOF, grip strength) were assigned to the body functions and structure component in the ICF model. Although some parameters from other components (especially activities and participation) showed close correlations with mobility, they could not show significant predictive value in the regression models. These results suggest that of the three components of functioning and disability, body functions and structures can be assessed most accurately by the commonly used instruments of the comprehensive geriatric assessment. This may be due to the fact that parameters from this component are most commonly recorded in the clinic, while activities and participation, on the other hand, mostly take place in the patient’s home environment. Thus, self-reported outcomes are often used for their assessment, which are prone to bias. In our model, the Barthel index was the only parameter from the activities and participation components that is not self-reported but assessed by nursing staff. This parameter showed the greatest association with mobility in all models within the mentioned components. These results argue in favor of including quantitative assessment approaches that measure the actual performance of patients in their home environment [64–66]. Current research, for example the Innovative Medicines Initiative (IMI) projects IDEA-FAST and Mobilise-D, deal with the validation of digital outcomes to assess mobility in home environment [67]. Further research should focus on developing such objective and performance-based assessment instruments that allow a holistic view on geriatric patients’ health problems and impairments.

### Limitations

A first limitation is the cross-sectional design, which makes it difficult to draw conclusions about the causality of the relationships. We addressed this by interpreting the relationship between the parameters cautiously in the discussion. Second, the interpretation of the associations is complicated by the fact that this is a multimorbid cohort with many functional limitations, which might also be affected by the admission diagnosis. To address this, we considered as many mobility-related parameters as possible and included them in post-hoc analyses, which did not add relevant aspects to the presented results. Third, we recruited participants from a specialist ward setting. Therefore, the associations cannot be applied to other clinical groups or the community dwelling general population. Finally, to assess mobility under realistic conditions, we included participants with and without walking aids. This makes interpretation of the data more complex, although the SPPB is validated for both conditions [14].

## Conclusions

Our cross-sectional analysis of an inpatient cohort of older adults shows that FOF and grip strength are associated with mobility in hospitalized geriatric patients. In particular, FOF appears to be a risk factor for mobility impairment such as gait, static balance, and transfer deficits. Prevention and treatment of FOF (also independent of actual falls) should therefore be an objective of future longitudinal studies, as should be improvement of muscle strength. Furthermore, these analyses suggest, at least indirectly, that (home-based) objective performance assessment instruments may add relevant information to our understanding of mobility deficits in geriatric patients.

## Data Availability

The datasets used and analysed during the current study are available from the corresponding author on reasonable request.

## Abbreviations

ADL: Activities of daily living
ComOn: COgnitive and Motor interactions in Older populatioNs
DIA-S: *Depression-im-Alter* scale
FES-I: Falls Efficacy Scale-International
FOF: Fear of falling
IADL: Instrumental activities of daily living
ICD: International Statistical Classification of Diseases and Related Health Problems
ICF: International Classification of functioning, disability and health
MDS-UPDRS: Movement Disorder Society-revised Unified Parkinson’s Disease Rating Scale
MoCA: Montreal Cognitive Assessment
NAA: *Nürnberger-Alters-Alltagsskala*
PD: Parkinson’s disease
SPPB: Short Physical Performance Battery
WHO: World Health Organization
